# Ultra-Smooth Polishing of Single-Crystal Silicon Carbide by Pulsed-Ion-Beam Sputtering of Quantum-Dot Sacrificial Layers

**DOI:** 10.3390/ma17010157

**Published:** 2023-12-27

**Authors:** Dongyang Qiao, Feng Shi, Ye Tian, Wanli Zhang, Lingbo Xie, Shuangpeng Guo, Ci Song, Guipeng Tie

**Affiliations:** 1 College of Intelligence Science and Technology, National University of Defense Technology, Changsha 410073, China; dyqiao@nudt.edu.cn (D.Q.); tianyecomeon@sina.cn (Y.T.); zhangwanli17@nudt.edu.cn (W.Z.); lingbotse@163.com (L.X.); g18231033287@163.com (S.G.); songci@nudt.edu.cn (C.S.); tieguipeng@163.com (G.T.); 2 Hunan Key Laboratory of Ultra-Precision Machining Technology, Changsha 410073, China; 3 Laboratory of Science and Technology on Integrated Logistics Support, National University of Defense Technology, 109 Deya Road, Changsha 410073, China; 4 Precision Optical Manufacturing and Testing Center, Shanghai Institute of Optics and Fine Mechanics, Chinese Academy of Sciences, Shanghai 201800, China

**Keywords:** single-crystal SiC, quantum dots, ultra-smooth polishing, pulsed-ion-beam, sacrificial layer

## Abstract

Single-crystal silicon carbide has excellent electrical, mechanical, and chemical properties. However, due to its high hardness material properties, achieving high-precision manufacturing of single-crystal silicon carbide with an ultra-smooth surface is difficult. In this work, quantum dots were introduced as a sacrificial layer in polishing for pulsed-ion-beam sputtering of single-crystal SiC. The surface of single-crystal silicon carbide with a quantum-dot sacrificial layer was sputtered using a pulsed-ion beam and compared with the surface of single-crystal silicon carbide sputtered directly. The surface roughness evolution of single-crystal silicon carbide etched using a pulsed ion beam was studied, and the mechanism of sacrificial layer sputtering was analyzed theoretically. The results show that direct sputtering of single-crystal silicon carbide will deteriorate the surface quality. On the contrary, the surface roughness of single-crystal silicon carbide with a quantum-dot sacrificial layer added using pulsed-ion-beam sputtering was effectively suppressed, the surface shape accuracy of the Ø120 mm sample was converged to 7.63 nm RMS, and the roughness was reduced to 0.21 nm RMS. Therefore, the single-crystal silicon carbide with the quantum-dot sacrificial layer added via pulsed-ion-beam sputtering can effectively reduce the micro-morphology roughness phenomenon caused by ion-beam sputtering, and it is expected to realize the manufacture of a high-precision ultra-smooth surface of single-crystal silicon carbide.

## 1. Introduction

The single-crystal silicon carbide (SiC) is a highly promising third-generation semiconductor material with excellent physical and mechanical properties such as large bandgap, high thermal conductivity, high specific stiffness, low coefficient of thermal expansion, and good abrasion resistance [1,2]. It has been widely used in high frequency, high temperature, radiation resistance, photoelectric, and other fields. A smooth surface with low damage is an inevitable requirement for the application of single-crystal silicon carbide. However, due to its high brittleness, hardness, and low fracture toughness, defects such as rough surfaces and microcracks are inevitably formed during the surface processing of SiC [3,4]. Achieving high-precision single-crystal silicon carbide manufacturing with an ultra-smooth surface is still a great challenge.

Polishing is the last step of single-crystal silicon carbide processing, and its surface quality directly affects the performance of semiconductor materials [5]. The chemical mechanical polishing (CMP) method is currently a typical method for preparing SiC [6]. However, due to the high chemical inertness of the surface of single-crystal silicon carbide, its etching efficiency is low. In addition, the process consumes a large amount of slurry and introduces impurity pollution [7], and the CMP method does not have a polishing ability for optical elements of free-form surfaces. Ion-beam sputtering (IBS) is based on the physical sputtering effect, using ions with a certain energy to bombard the surface of the optical element to achieve atomic-level material removal, which is less affected by the chemical properties and hardness of the element. IBS can polish complex free-form surfaces without inducing subsurface damage [8]. In order to improve the machining accuracy of optical components of high-hardness materials, F. Shi et al. proposed a pulsed-ion-beam machining technology with sub-nanometer machining accuracy based on traditional ion-beam machining [9,10,11]. In light of the evolution of the surface morphology of ion-beam sputtering elements, researchers have conducted many experimental and theoretical studies. Allen [12] studied the surface roughness evolution of ion-beam polishing fused silica, and the results showed that the surface roughness value increased with an increase in the removal depth. At the same time, experiments have also shown that ion-beam sputtering can effectively reduce surface roughness [13,14]. Bradley and Harper established the linear evolution theory (BH model) of surface micro-topography based on the Sigmund sputtering theory. They pointed out that the local etching rate is related to the local curvature, and the energy deposited in the local pits is more than that in the bulge, so the etching rate of the pits is greater than that of the bulge, resulting in the roughening of the surface micro-topography [15]. At the same time, the thermally induced surface diffusion effect and surface porosity mechanism make the ion sputtering have a smoothing effect on the surface [16,17]. Due to the uncertainty of the ion beam smoothing the surface of optical elements, the IOM Institute [18,19] proposed a sacrificial layer-assisted polishing method. A material layer such as photoresist, silicon, and SiO_2_ is uniformly covered on the initial surface by coating or sputtering deposition, and then the material is smoothed directly using ion-beam sputtering until an ultra-smooth surface is obtained.

At first, quantum dots were introduced into the machining process as a subsurface damage detection method. K.L.M Williams et al. [20] used a nano-scale quantum dot solution to label the subsurface defects formed in the grinding and polishing process. The depth of the subsurface defect layer was calibrated via fluorescence labeling. M. Chen et al. [21] showed that improving the size distribution of HgSe quantum dots could exponentially increase their mobility, and it is necessary to improve the size distribution when using in-band photodetectors. Benjamin T et al. proved that CdSe nanosheet quantum wells had a narrow, polarized intersubband and absorption characteristics under light excitation or external bias [22]. Xin Tang et al. fabricated functional quasi-3D nanophotonic structures directly into colloidal quantum dot (CQD) films using the one-step imprinting method, and the diffraction gratings, double-layer wire-grid polarizers, and resonant metal mesh long-pass filters were imprinted on the CQD films [23]. These studies show that quantum dots exhibit large specific surface areas with excellent optical, electrical, and thermal properties due to the quantum confinement effect and boundary effect and can be used as sacrificial layer materials to introduce the polishing process of optical elements.

This paper aimed to study the roughness evolution of single-crystal SiC surfaces using pulsed-ion-beam sputtering and to realize the ultra-smooth machining of single-crystal SiC surfaces. Through experimental research and theoretical analysis, we found that adding a quantum dot coating as a sacrificial layer on single-crystal silicon carbide could hinder the roughening phenomenon caused by different sputtering characteristics of traditional ion beam bombardment of dual-phase materials and achieve high-precision modification of single-crystal silicon carbide while obtaining a higher surface quality. We anticipate that this method will apply to industrial-scale ultra-smooth polishing of SiC.

## 2. Experimental Setup

### 2.1. Material

**Figure 1:**
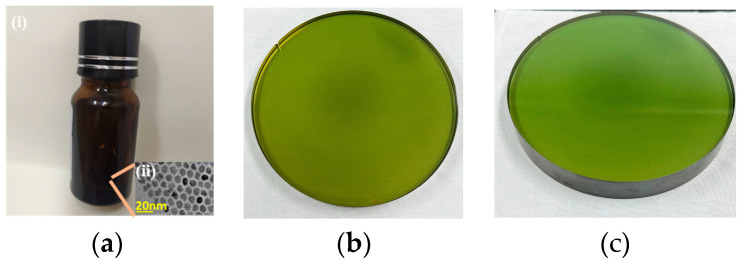
Materials used in the experiment. (**a**) CdSe/ZnS quantum dot solution. (i) Reagent bottle containing quantum dot solution. (ii) Transmission electron micrograph of a quantum dot. (**b**) Single-crystal silicon carbide sample 1. (**c**) Single-crystal silicon carbide sample 2.

This study used two 4H-SiC lenses provided by TankeBlue Semiconductor Co. Ltd., Beijing, China, 120 mm in diameter and 10 mm in thickness. All experiments were carried out on the most commonly used Si (0001) surface of electronic devices, and the samples are shown in Figure 1b,c. First, all samples were ultrasonically cleaned with deionized water (conductivity < 0.5 us/cm) for 30 min, then dehydrated and dried with anhydrous ethanol. Deionized water and anhydrous ethanol were obtained from Aladdin Scientific Corp. In this study, we used a commercially available (Mesolight Co. Ltd., Suzhou, China) water-soluble CdSe/ZnS core–shell structured quantum dot solution with a luminescence wavelength of 544 nm ± 10 nm (Figure 1a).

### 2.2. Pulsed-Ion-Beam Etching Equipment

**Figure 2:**
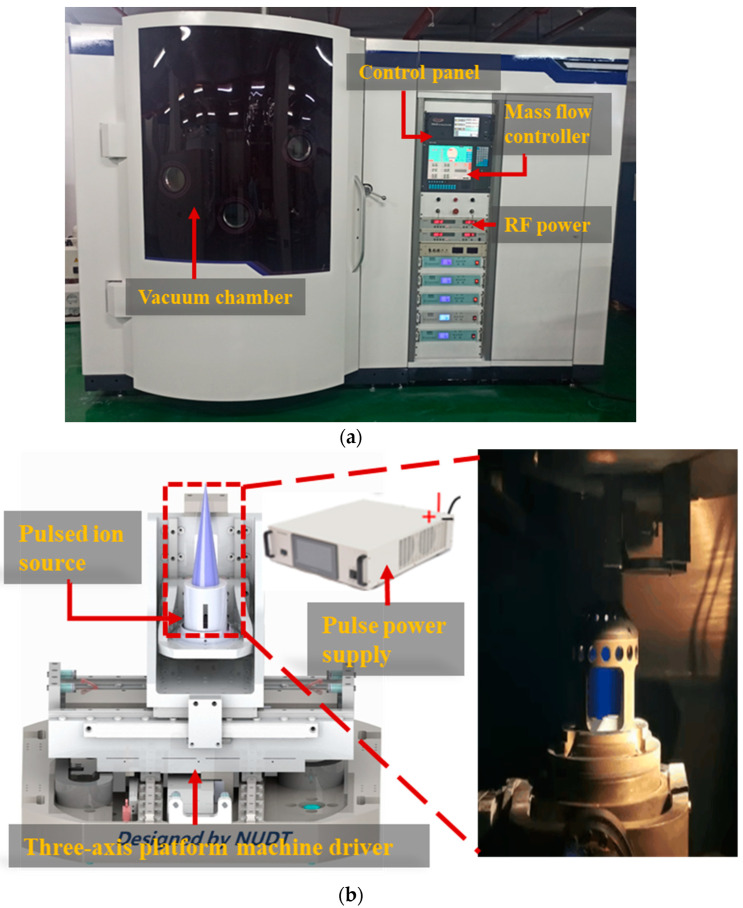
Physical diagram and schematic diagram of the pulsed-ion-beam etching equipment. (**a**) NUDT-IBE700 equipment. (**b**) Pulsed ion source model schematic diagram and pulsed ion source device physical diagram.

The process of pulsed-ion-beam etching of single-crystal silicon carbide was carried out on the ion-beam etching machine shown in Figure 2a. Argon (Ar) is ionized into Ar^+^ in the ion source, forming a plasma in the cavity. The three-grid ion optical system accelerated and focused the ion beam, which was then energized using the accelerated electric field to form material removal on the surface of the workpiece.

As shown in Figure 2b, a highly controllable high-voltage pulse power supply was connected to the screen grid of the ion optical system. The high-voltage pulse power supply supplied power to the screen grid, and the extraction method of the ion beam was changed from continuous extraction to pulsed extraction. The continuous ion beam was also intercepted as a pulsed ion beam. The self-developed pulsed ion-beam processing equipment used argon as the etching gas, and its pulse frequency was adjusted in the range of 1–500 Hz. The pulse ion-beam removal resolution can reach 0.07 nm in a single shot, achieving the optical mirror’s atomic level removal accuracy.

### 2.3. Method

The experiment was carried out in a self-developed IBE system. The working pressure was 2.5 × 10^−3^ Pa, the Ar^+^ ion energy was 800 eV, the beam density was 10–25 mA, and the working temperature was 70 °C.

**Figure 3:**
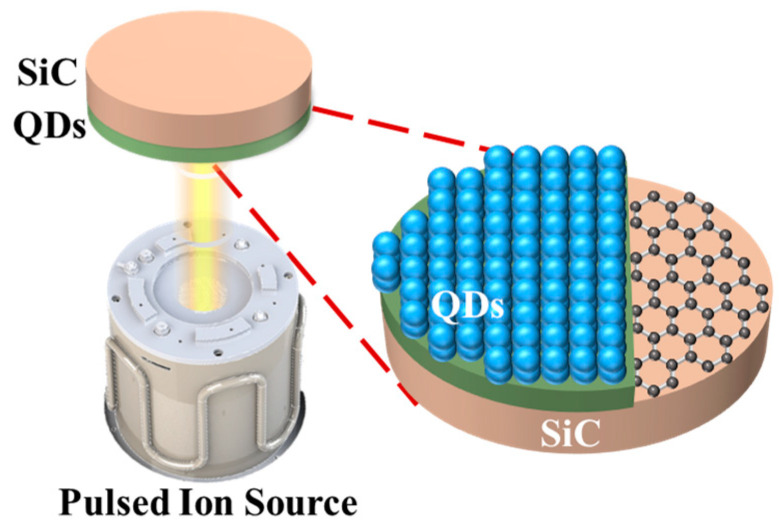
Quantum-dot sacrificial layer pulsed-ion-beam etching method.

**Figure 4:**
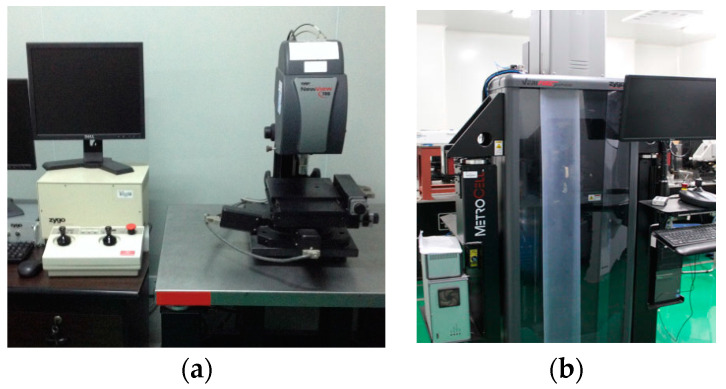
Measurement equipment. (**a**) Zygo white light interferometer. (**b**) Zygo wavefront interferometer.

The surface of sample 1 was left untreated. A layer of quantum dots was coated on the surface of single-crystal silicon carbide sample 2 using the spin-coating method, in which a quantum dot solution was dropped on the surface of the substrate. Then, the substrate was rotated to allow the quantum dots to cover the surface uniformly by centrifugal force, and then the etching and polishing research was carried out under the same etching parameters. The same pulse frequency was maintained during the pulse-ion-beam etching and polishing process to ensure the stability of the removal function. The etching process is shown in Figure 3. In order to observe the evolution of surface quality, the white light interferometer (Figure 4a) was used to observe the surface morphology of single-crystal silicon carbide. A commercial Zygo New View 700 s white light interferometer was used to detect the medium and high frequencies of the micron scale on the wafer surface. The lens was equipped with magnifications of 10× and 50×, and the resolution of the data was 1.5 μm and 7.5 μm, respectively. The detection range was 468 μm × 351 μm. The VeriFire Asphere laser wavefront interferometer by Zygo was used to detect low-frequency surface errors, as shown in Figure 4b. The maximum aperture of the interferometer standard lens was 150 mm, and the maximum resolution was 1024 × 1024 pixels.

## 3. Results and Discussion

**Figure 5:**
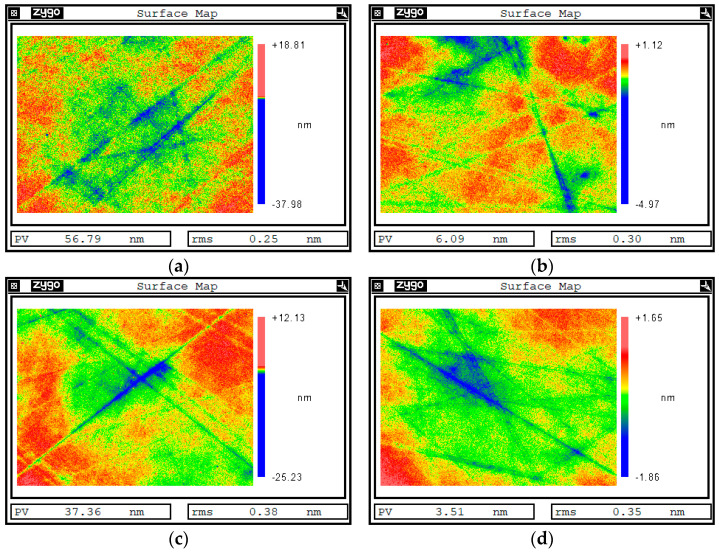
Detection results of white light interferometer on the surface of sample 1. (**a**) Surface area a of the sample before sputtering. (**b**) Surface area b of the sample before sputtering. (**c**) Surface area a of the sample after sputtering. (**d**) Surface area b of the sample after sputtering.

**Figure 6:**
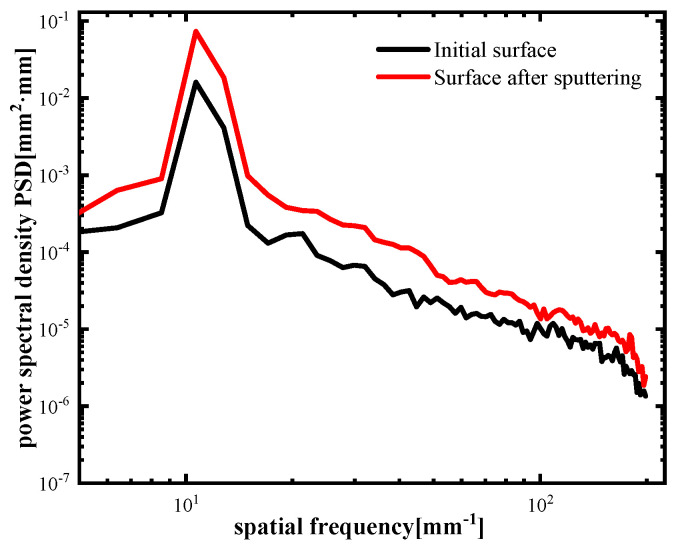
The PSD curves of the initial surface without quantum dots and after IBE polishing.

Figure 5 shows the change in surface roughness of single-crystal silicon carbide without a quantum-dot sacrificial layer under common etching parameters (ion energy of 800 eV, beam density of 20 mA, and duty cycle of 50%). Two points were taken on the surface of the original sample to measure the initial roughness. PV (peak to valley) is the difference between the highest and lowest part of the surface, and RMS (root mean square) is the root mean square value. The original surface roughness was 0.25 nm RMS (Figure 5a) and 0.30 nm RMS (Figure 5b). After ion-beam etching for 30 nm, the surface roughness of the same area became 0.38 nm RMS (Figure 5c) and 0.35 nm RMS (Figure 5d). The surface roughness of single-crystal silicon carbide increased, and the surface quality deteriorated. According to the results of the PSD curve (Figure 6), the error of medium- and high-frequency bands was worse than that of the initial surface after ion-beam sputtering on the surface of single-crystal silicon carbide, which shows that there is a rough effect on the surface of single-crystal silicon carbide directly bombarded by the ion beam.

**Figure 7:**
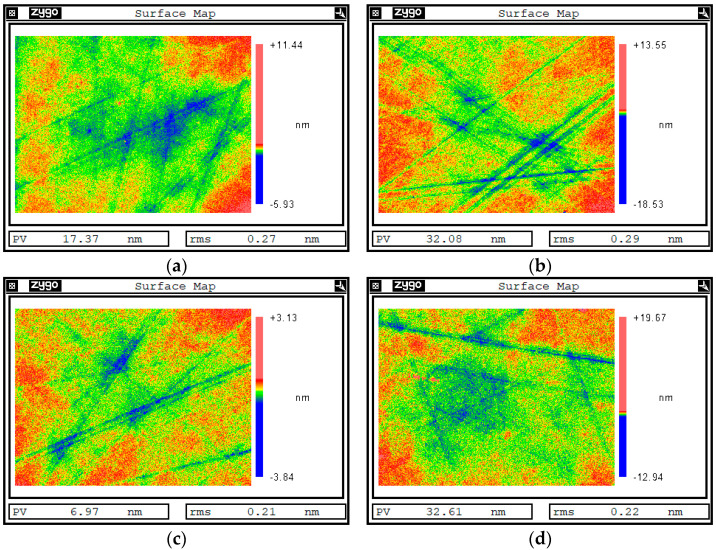
Surface white light interferometer test results of sample 2 with a quantum-dot sacrificial layer. (**a**) Surface area a of the sample before sputtering. (**b**) Surface area b of the sample before sputtering. (**c**) Surface area a of the sample after sputtering. (**d**) Surface area b of the sample after sputtering.

**Figure 8:**
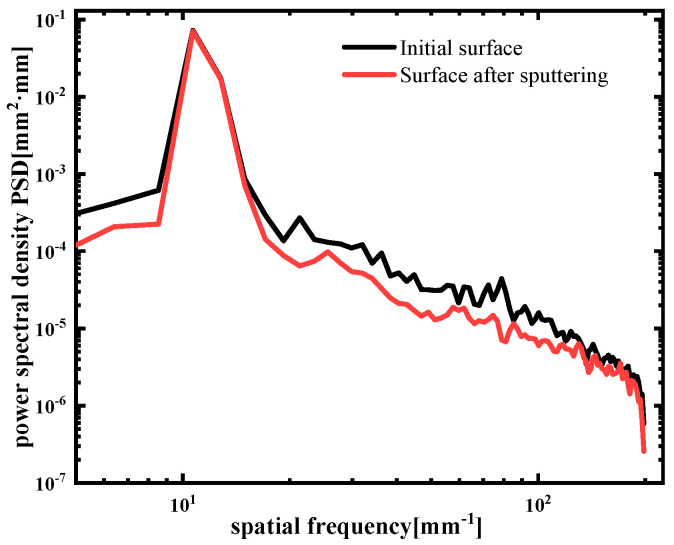
The PSD curves of the initial surface with quantum dots and after IBE polishing.

Under the same etching parameters (ion energy of 800 eV, beam current size of 20 mA, and duty cycle of 50%), 30 nm was etched on the surface of single-crystal silicon carbide with a sacrificial layer. In contrast, the experimental results in Figure 7 show that the surface quality was effectively improved by adding quantum dots as sacrificial layers after pulsed-ion-beam removal. The original surface roughness of the sample was 0.27 nm RMS (Figure 7a) and 0.29 nm RMS (Figure 7b). After ion-beam etching, the surface roughness of the same area became 0.21 nm RMS (Figure 7c) and 0.22 nm RMS (Figure 7d). According to the PSD curve (Figure 8), the results showed that the full-band curve of the surface after pulsed-ion-beam sputtering was lower than that of the initial surface. After adding the quantum-dot sacrificial layer to the surface of single-crystal silicon carbide, pulsed-ion-beam sputtering could improve the surface quality.

The roughness and smoothing effect interaction dominates the morphology change with bombardment time in ion sputtering. For any microscopic morphology $m\left(x,y,t\right)$ combining partial differential equations and statistical methods, the change in surface morphology m(q,t) and in frequency space with time can be expressed as:

(1) $$\frac{\partial m(q,t)}{\partial t}=-m\left(q,t\right)M\left(q\right)+\eta \left(q,t\right)$$

For the statistical linear equation of Equation (1), Moselerp [24] and Spiller et al. [25] derived the relevant solutions and obtained the power density function:

(2) $$PSDm\left(q,t\right)=PSD\left(q,t=0\right)\exp\left(2M\left(q\right)t\right)+A\frac{1-\exp\left(2M\left(q\right)t\right)}{M\left(q\right)}$$

M(q) depends on the processing parameters of ion-beam sputtering. The $q^{n}$ component in the polynomial represents different sputtering roughness and smoothing processes. *t* is the introduced sacrificial layer parameter, namely:

(3) $$M\left(q\right)=\left(M_{2s}+M_{2B}\right)q^{2}-{t\times M}_{4f}q^{4}$$

Therefore, it can be seen that the evolution of surface micro-morphology is the result of the combined action of surface roughness and smoothness, and different processing conditions determine the development trend of surface morphology. If the effect of viscous flow and elastic diffusion is dominant, M(q) is negative, and the surface develops in a smooth direction. Otherwise, M(q) is positive, and the surface will become rougher. The root mean square roughness *Rq* of the microstructure can be obtained as follows:

(4) $$Rq=2\pi \int_{0}^{\infty }PSD\left(q,t\right)qdq$$

According to the above theoretical research, the evolution process of the ion-beam etching of single-crystal silicon carbide was analyzed, and the change in surface roughness under ion beam bombardment was obtained.

The process of ion-beam sputtering of the single-crystal silicon carbide surface has both surface smoothing and rough effects. The experimental results in Figure 5 also verified this theory. When the effect of viscous flow and elastic diffusion on the surface of sputtered single-crystal silicon carbide cannot eliminate the influence of the roughness effect, the roughness effect plays a leading role, the surface quality will deteriorate, and the roughness will increase, as shown in Figure 5c,d. RMS increased to 0.38 nm and 0.35 nm. The PSD curve in Figure 6 also shows that the error in the surface’s middle- and high-frequency bands after ion-beam sputtering deteriorated.

The quantum-dot sacrificial layer added to the surface of single-crystal silicon carbide made the *t* × $M_{4f}$ part negative, while the front positive value was very small compared to the change in t. It can be seen that $M\left(q\right)$ always remained negative, and the surface of the single-crystal silicon carbide always developed in a smooth direction. The results in Figure 7 also show that the roughness of the surface was reduced after adding the quantum dot solution as the sacrificial layer, and the minimum RMS could reach 0.21 nm, which realizes the ultra-smooth surface manufacturing of single-crystal silicon carbide. The PSD curve in Figure 8 also shows that the surface’s middle- and high-frequency bands greatly improved after adding a quantum-dot sacrificial layer via ion-beam sputtering on the surface.

**Figure 9:**
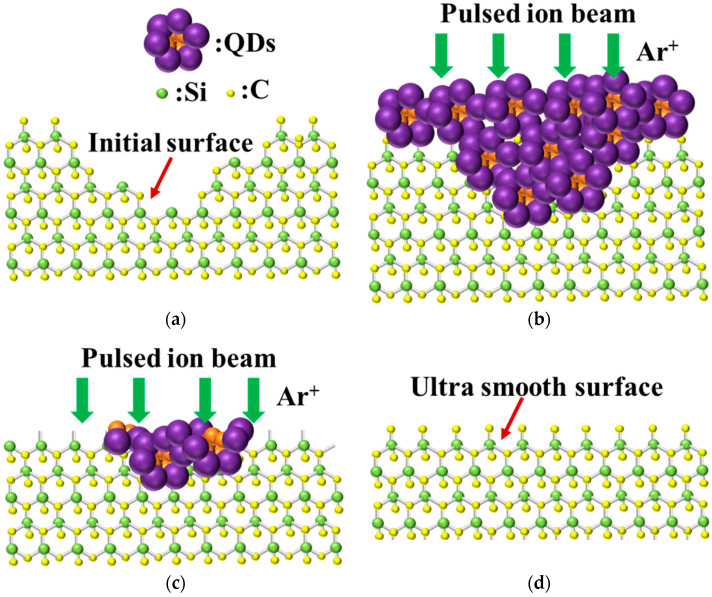
Ion-beam sputtering removal with a sacrificial layer. (**a**) Initial surface of single-crystal silicon carbide. (**b**) Surface after adding the quantum-dot sacrificial layer. (**c**) Polishing process. (**d**) Ultra-smooth surface of single-crystal silicon carbide.

Figure 9 demonstrates the pulsed-ion-beam sputtering of a single-crystal SiC surface with a sacrificial layer of quantum dots. Quantum dots were added to the rough initial surface, as shown in Figure 9b; the added quantum dots filled the surface scratches and pits and made the surface flat. Then, the surface was etched using a pulsed ion beam to remove the added quantum-dot sacrificial layer, as shown in Figure 9c. As the pits were filled, the difference in the energy deposition and etching rate at various ion-beam sputtering pits was suppressed. Thus, the surface was uniformly removed, and after the quantum-dot sacrificial layer was completely removed, an ultra-smooth surface was obtained, as shown in Figure 9d.

**Figure 10:**
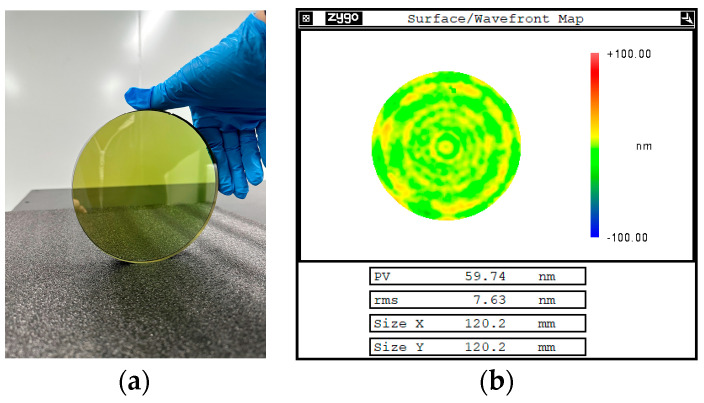
Pulsed-ion-beam sputtering of quantum-dot sacrificial layer single-crystal silicon carbide processing examples. (**a**) Physical drawing of the processing sample. (**b**) Surface shape results after processing.

The quantum-dot sacrificial layer was coated on the surface of the single-crystal silicon carbide with an aperture of 120 mm, and the whole surface was modified by the pulsed ion beam. After polishing, the surface shape accuracy converged to 7.63 nm RMS (Figure 10), and the roughness was reduced to 0.21 nm RMS, which realizes the high-precision ultra-smooth polishing of single-crystal silicon carbide.

## 4. Conclusions

This paper proposed a pulsed-ion-beam ultra-smooth polishing method for single-crystal SiC by introducing quantum dots as sacrificial layers. A water-soluble CdSe/ZnS core–shell quantum dot solution was used to coat the surface of the single-crystal silicon carbide, and then pulsed-ion-beam sputtering etching was carried out at an ion energy of 800 eV and a beam density of 20 mA. An ultra-smooth surface with a roughness of 0.21 nm RMS was fabricated. The main conclusions of this paper are as follows:

(1) Pulsed-ion-beam etching based on the material removal mechanism by physical sputtering can be used as a high-precision and low-damage process to achieve ultra-smooth polishing of high-hardness single-crystal silicon carbide;
(2) Due to energy deposition and thermal diffusion, the surface roughness of single-crystal silicon carbide surfaces with different initial morphology can be increased by ion-beam sputtering, and the surface quality can deteriorate. This phenomenon can be attributed to the coexistence of the smoothing and roughening effects in ion-beam sputtering surface material;
(3) The introduction of quantum dots as a sacrificial layer can change the energy deposition distribution and etching rate to increase the surface smoothing effect of pulsed-ion-beam sputtering and realize the ultra-smooth surface polishing of the single-crystal silicon carbide surface. The experimental results showed that the surface shape accuracy of the quantum-dot sacrificial layer after pulsed-ion-beam sputtering etching was 7.63 nm RMS, and an ultra-smooth surface of single-crystal silicon carbide with a roughness of 0.21 nm RMS was realized.

In summary, the introduction of quantum dots as a sacrificial layer in the process of pulsed-ion-beam etching to polish single-crystal silicon carbide can improve the surface quality, which provides theoretical and technical support for the acquisition of an ultra-smooth surface of single-crystal silicon carbide, and also provides a new idea for the ultra-smooth polishing of high-hardness optical components.

## Data Availability

The data presented in this study are available on request from the corresponding author. The data are not publicly available because the data are also part of an ongoing study.
